# The impact of rural revitalization talent cultivation on farm household part-time farming: Evidence from the “One Village, One University Student” program

**DOI:** 10.1371/journal.pone.0318680

**Published:** 2025-02-12

**Authors:** Jiawei Wang, Xia Kuang, Zhipeng Wang, Wenmei Liao, Hailan Qiu

**Affiliations:** 1 School of Economics, Jiangxi University of Finance and Economics, Nanchang, China; 2 Digital Faculty of Economics, Jiangxi Open University, Nanchang, China; 3 School of Economics and Management, Jiangxi Agricultural University, Nanchang, China; Universitas Airlangga, INDONESIA

## Abstract

The key to rural revitalization lies in talent revitalization. Cultivating a workforce that understands agriculture, loves rural areas, and cares for farmers, while retaining talent in rural regions, is of greater significance for comprehensively advancing rural revitalization. From the perspective of farm household part-time farming, this study takes the *One Village, One University Student* program as an example. Using survey data from 2552 farm households across 12 counties (cities and districts) in Jiangxi Province, China, and employing the ESR model, this study empirically analyzes the impact of rural revitalization talent cultivation on farm household part-time farming. It further explores the differentiated effects of various educational levels and learning modes of talent cultivation on the part-time farming. The results show that rural revitalization talent cultivation significantly reduces the degree of part-time farming, with higher-level education and correspondence education having particularly pronounced effects in enhancing agricultural skills and production efficiency. Based on these findings, this paper proposes policy recommendations to intensify rural revitalization talent cultivation, improve the educational levels and content, optimize educational modes, and strengthen the policy advocacy, aiming to further promote rural economic development and increase farmers’ income.

## Introduction

Since 1984, when China first proposed that the land contract period should remain unchanged for a long time, the relationship between people and land, which has accumulated over the past 40 years, has become very complicated, resulting in the existence of many phenomena, such as “the deceased have land to plant, the living do not have land to cultivate, the relocated population leaves land barren, and the newly added populations has no land to hope for” [1]. Moreover, with the rapid development of China’s industrial and service sectors, the opportunity cost of agricultural labor has risen steadily [2]. As a result, under the constraints of China’s relatively acute conflict between land and people and the inadequacy of social security in the countryside, the phenomenon of part-time farming has become increasingly common; in other words, to maximize the returns of the family, farmers’ livelihood choices are a combination of agricultural production and nonagricultural employment [3]. Part-time farming has long been recognized as a global phenomenon [4]. It is a common feature of agricultural development worldwide, particularly during the process of industrialization. For example, in Germany, Norway, Austria and Switzerland, the rate of part-time farming exceeds 50%, whereas in Asia, it exceeds 80% in Japan, South Korea and Taiwan. Part-time farming has become the most prominent socioeconomic phenomenon in China’s rural areas, and over the years of rural reform, the rate of part-time farming has remained high.

Therefore, what factors lead farm households to choose part-time farming? Existing studies have primarily analyzed this topic from two perspectives. First, from a macro perspective, research has focused on the external driving factors and environmental conditions influencing farm households’ engagement in part-time farming. One reason farm households engage in part-time farming is to cope with the instability of agricultural income [5]. That is, the pursuit of maximizing returns is the internal driving force for the formation of part-time farming behavior. Moreover, the process of urbanization and changes in the rural labor market are important factors that motivate farm households to engage in part-time farming [6]. Changes in agricultural production conditions, such as reductions in land resources and climate change, are also considered important factors affecting the part-time farming behavior [7]. Second, from a micro perspective, studies have examined the influence of household-level factors on part-time farming behavior, focusing on how various internal household factors shape these decisions. Research has shown that household members with higher education levels are more inclined to participate in non-agricultural employment to increase total household income [8]. In addition, the asset status of the household, such as the size of the land owned and the means of production, also affects the need to increase income through part-time farming [9]. Most of these studies emphasize the impact of external conditions and household factors on farm households’ part-time farming behavior. Only a small number of studies have examined this behavior from the human capital perspective [10]. However, there is a lack of further analysis on the differential effects of enhancing farmers’ agricultural human capital on agricultural and nonagricultural employment.

Part-time farming refers to the practice of farm households combining agricultural production with non-agricultural employment as a strategy to increase household income and diversify agricultural risks. It represents not only an adaptive response to the inherent uncertainties of agricultural income but also an inevitable outcome of the diversified development of rural labor markets. However, while part-time farming is conditionally recognized as a rational livelihood strategy in certain contexts, mainstream views often regard it as a transitional form [11,12]. Specifically, it is seen as a temporary phase in the broader shift from traditional smallholder agriculture to more specialized, large-scale farming or complete non-agricultural employment. In the long run, the trend of part-time farming may lead to the dispersal of agricultural production resources, thus hindering improvements in agricultural production efficiency. Consequently, greater emphasis should be placed on the transition of agriculture towards scale and specialization [13]. In fact, Chinese farm households are currently evolving in two directions: nonagricultural part-time households and specialized large-scale farming households. In 2002, China enacted the *Rural Land Contract Law*, which stipulated that “contractors have the right to independently decide whether and how to transfer contracted land management rights in accordance with the law” and specified detailed transfer methods such as subcontracting, leasing, swapping, and transferring. Furthermore, the 2023 *No. 1 Central Document* noted that “the basis of collective land ownership in rural areas should be strengthened, the contracting rights of farmers should be stabilized, and the land management rights should be further revitalized”. It actively guiding part-time farming households to participate in land transfers to address institutional barriers in the farmland system. Against this backdrop, cultivating and developing moderately scaled specialized farm households has created new opportunities for agricultural development. In 2004, the Ministry of Education launched the *One Village, One University Student* program (hereinafter referred to as the OVOUS program). This initiative aims to cultivate new agricultural management entities such as large-scale professional households, family farms, professional farmer cooperatives, and industrialized leading enterprises, actively promoting the development of various forms of large-scale management.

Therefore, with the steady advancement and continuous improvement of the “three rights separation” system of rural land, it remains an urgent task to further promote the principle of “ capable individuals engaging in farming”. Therefore, this study takes the OVOUS program as an example to explore the impact of rural revitalization talent cultivation on farm households’ part-time farming. It analyzes the differential effects of various academic levels and learning modes of talent cultivation on part-time farming and proposes corresponding policy recommendations to promote rural talent revitalization.

## Theoretical analysis and research hypotheses

### The impact of rural revitalization talent cultivation on farm households’ part-time farming

The common concept of part-time farmers refers to individuals who are forced to seek additional employment due to low or declining agricultural income—often described as “failed full-time farmers” [14]. Rural revitalization talent cultivation, however, has made significant contributions to reducing the degree of part-time farming among farm households. The detailed analysis is as follows. First, improving agricultural productivity and profitability. Talent cultivation programs in rural revitalization usually include modern agricultural technology training, agricultural product processing and marketing skills upgrading. These improvements help farm households increase efficiency and quality in agricultural production, leading to higher yields and income [15]. When agricultural production becomes more profitable, farm households are more likely to focus on their primary agricultural activities rather than seeking additional non-agricultural employment to supplement household income. Second, enhancing professional skills and competitiveness. Rural revitalization talent cultivation is not limited to agricultural skills but also emphasizes the improvement of overall competencies [16], such as management skills, legal knowledge and financial knowledge. These advancements enhance the competitiveness of farm households in the rural market economy, enabling them to manage their agricultural operations more efficiently, increase their incomes, and reduce dependence on external part-time job. Third, promoting rural industrial integration. Rural revitalization talent cultivation helps farm households understand and participate in the integration of rural industries, such as combining agriculture with tourism, culture, health industries. This diversified development not only broadens the scope of farm households’ operations but also creates additional income opportunities [17,18]. Through industrial integration, more local employment opportunities are generated, encouraging farm households to remain in the rural areas and engage in various forms of production activities instead of seeking work elsewhere.

Overall, rural revitalization talent cultivation enhance the skills and income potential of farm households, thereby reducing their reliance on non-agricultural part-time jobs. This enables them to focus more on primary agricultural activities and the development of the rural economy, effectively lowering the degree of part-time farming. Based on the above analysis, the following research hypothesis is proposed:

H1: Rural revitalization talent cultivation significantly reduce the degree of part-time farming among farm households.

### The impact of rural revitalization talent cultivation at different educational levels on farm households’ part-time employment

In the context of rapid technological changes, the impact of education on agricultural productivity has become increasingly significant, as it enables farm households to better adapt new opportunities brought by technological innovation [19]. Participation in higher-level educational programs is more effective in reducing the degree of part-time farming among farm households. The specific analysis is as follows. First, improving agricultural skills and efficiency. Higher-level education and training provide more advanced agricultural knowledge and skills, such as innovative planting techniques, pest and disease management, and soil improvement. This specialized knowledge can significantly enhance production efficiency and the quality of agricultural products [20]. As a result, higher agricultural income reduces the need to seek non-agricultural income sources, thereby lowering the degree of part-time farming. Second, enhancing innovation and adaptability. Higher-level education typically includes training in innovative thinking and a broad knowledge base, which helps farm households better respond to market changes and environmental challenges, improving agricultural productivity [21]. Through education and training, farm households can more flexibly adjust their production methods and adopt new agricultural technologies and management models, thereby stabilizing incomes and reducing dependence on external non-agricultural income. Third, improving understanding of policies and markets. Higher-level education enables farm households to better leverage government agricultural support policies, such as subsidies, loans and technical support programs. It also enhances their ability to understand market trends and develop effective business strategies. This sensitivity to policies and markets can significantly improve the stability and predictability of agricultural incomes [22].

Based on the above analysis, the following research hypothesis is proposed:

H2: Participation in higher-level educational programs in talent cultivation is more effective in reducing the degree of part-time farming among farm households.

### The impact of different learning modes of rural revitalization talent cultivation on farm households’ part-time farming

Corresponding education holds a prominent position in countries with large, dispersed rural populations [23]. Participation in correspondence education is often more effective than distance education in reducing the degree of part-time farming. The specific analysis is as follows. First, learning experience and depth of knowledge acquisition. Correspondence education typically involves systematic materials and regular face-to-face sessions, allowing farm households to gain a deeper understanding of complex agricultural knowledge and skills, which helps improve agricultural production efficiency. Although distance education is flexible and convenient, it primarily relies on online resources and the learner’s self-discipline. For participants with weaker self-learning abilities or poor time management, the effectiveness of distance education may be less comprehensive, impacting the practical application of knowledge [24]. Second, practical opportunities and skills applications. Corresponding education often includes practical courses or field experiments, enabling farm households to apply the knowledge learned in real-world setting. Such practice helps them master operational skills and translate theoretical knowledge into productivity. In contrast, distance education, due to its format limitations, often lacks opportunities for hands-on practice. While some courses offer virtual labs or online simulations, there cannot fully replicate real-world experiences. Additionally, may distance learning materials are described as monotonous, such as “dull gray professors reading dull gray notes on dull gray screens” [25]. Third, social interaction and networking building. The teacher‒student relationship in correspondence education often goes beyond the typical interaction seen in mass teaching [26]. This deeper engagement not only enhances academic understanding but also helps participants build valuable social networks. In comparison, teacher–student interaction in distance education is mostly asynchronous [27], and social engagement is often limited to online forums or video meetings. These forms of interaction lack the depth and immediacy of face-to-face communication, making them less effective.

Based on the above analysis, the following research hypothesis is proposed:

H3: Participation in correspondence education is more effective than distance education in reducing the degree of part-time farming among farm households.

## Data sources, indicator selection and model construction

### Data sources

As a major agricultural province, Jiangxi plays a pivotal role in China as one of the key rice-producing regions. To cultivate high-quality practical rural talents, Jiangxi Province launched the OVOUS program in 2012. This program adopts a combined training mode of correspondence and distance education, ensuring that participants receive vocational higher education locally or nearby through on-campus learning, alternating theory and practice, and teaching delivered to rural areas. The program primarily targets rural professional households, leaders of agricultural industrialization enterprises, and heads of rural cooperative organizations, particular those with college degrees under the national education system. The OVOUS program in Jiangxi Province is a key initiative for rural revitalization talent cultivation. Its goal is to enhance farmers’ educational qualifications and skills, improving their agricultural productivity and market competitiveness. Since the program’s implementation in Jiangxi Province, cumulative government funding at various levels has reached 450 million yuan, attracting over 82,000 participants with an average enrollment age of 33.6 years. The number of farmers receiving higher education has risen from an average of 0.4 per administrative village in 2012 to nearly 5 today. Jiangxi Province stands out as the province with the longest implementation period, the highest number of participants, the most advanced training levels, and the widest coverage under the Ministry of Education’s OVOUS program, providing robust talent support for Jiangxi’s rural revitalization. Therefore, Jiangxi Province serves as a representative and exemplary case study region.

To investigate the impact of rural revitalization talent cultivation on the degree of part-time farming among farm households, the research team conducted a telephone survey in December 2019. The experimental group consisted of farm households who had participated in the OVOUS project after 2012, while the control group included farm households who had not participated in the program. The survey followed a multi-stage sampling method: First, 12 counties in Jiangxi Province were randomly selected based on per capita industrial added value. Then, three townships were randomly chosen within each sample county, stratified by per capita public financial revenue. Within each townships, three administrative villages were randomly selected based on regional distribution. Finally, 10 non-participating farm households were randomly selected in each village. In total, 2,805 questionnaires were collected. After excluding those with missing key variables or major logical errors, 2,552 valid questionnaires were retained, resulting in an effective response rate of 90.98%.

### Indicator selection

#### 1. Explained variables.

The degree of part-time farming. This is measured as the proportion of agricultural income in total household income [28]. Part-time farming refers to the phenomenon where farm households, in pursuit of maximizing household utility, allocate part of their labor to non-agricultural sectors such as industry or services, thereby engaging in both agricultural and non-agricultural production.

#### 2. Core explanatory variables.

Rural revitalization talent cultivation. This study focuses on the impact of participating in the OVOUS program on the degree of part-time farming. Farm households that participated in the OVOUS program were assigned a value of 1, while non-participants were assigned a value of 0. Among the 2,552 respondents, 1,502 respondents had participated in the program, while 1,050 respondents had not.

#### 3. Control variables.

Drawing on existing studies, this paper incorporates control variables at the individual, household, and regional level. At the individual level, we focus on the characteristics of the household member who participated in the OVOUS program, variables include age, gender, education level, whether the individual has served as village cadres, and engagement in non-agricultural work. At the household level, variables include family size, whether the household belongs to the largest surname in the village, number of laborers, number of migrant works, membership in cooperatives, whether the household sells agricultural products online, whether the household has transferred farmland, and the actual cultivated land area. At the regional level, variables include the village terrain, distance to the county seat, and the regional economic development level.

#### 4. Instrumental variables.

Policy awareness. To address reverse causality and ensure the model identifiability, this paper introduced farm households’ awareness of the OVOUS program as an instrumental variable [29]. As an instrumental variable, it should causally influence farm households’ participation in the program without directly affecting the degree of part-time farming. After analyzing multiple variables, this paper determined that farmers’ awareness of the OVOUS program (hereinafter referred to as “policy awareness”) is likely to influence their decision to participate in the program but does not directly affect the degree of part-time farming. Therefore, “policy awareness” was selected as the instrumental variable. The definitions and descriptive statistics of these variables are detailed in Table 1.

**Table 1:** Variable definitions and descriptive analysis.

Variable type	Variable name	Definition and Assignment	Mean	SD
Explained variable	Degree of part-time farming	Proportion of agricultural income to total household income	0.156	0.283
Explanatory variable	Rural Revitalization Talent Cultivation	Participate in the OVOUS program: Yes = 1, No = 0	0.589	0.492
Individual characteristic	Age	Age of respondents	46.971	12.779
	Gender	Male = 1, Female = 0	0.757	0.429
	Educational level	Education level before participating in the OVOUS program: College degree or above = 5, Senior high school = 4, Vocational high school/secondary technic school = 3, Junior high school = 2, Primary school and below = 1	3.510	1.644
	Village cadres	Yes = 1, No = 0	0.442	0.496
	Migrant worker experience	Yes = 1, No = 0	0.650	0.477
Household characteristics	Family size	Total number of family members	4.796	1.802
	Largest surname in village	Yes = 1, No = 0	0.417	0.493
	Number of laborers	Number of family laborers	2.762	1.348
	Number of migrant workers	Number of family members working outside	1.472	1.381
	Cooperatives membership	Yes = 1, No = 0	0.164	0.370
	Online agricultural sales	Yes = 1, No = 0	0.085	0.279
	Farmland transfer in	Yes = 1, No = 0	0.446	0.497
	Cultivated land area	Actual cultivated land in area (hm^2^)	0.617	3.280
Regional characteristics	Village terrain	Mountains = 3, Hills = 2, Plains = 1	2.222	0.729
	Distance to county seat	Distance from village committee to county government (km)	27.105	20.624
	Regional economic development level	Per capita disposable income (10,000 yuan)	1.486	0.322
Grouping variable	Correspondence education participant	Yes = 1, No = 0	0.317	0.492
	Distance education participant	Yes = 1, No = 0	0.271	0.496
	College degree participant	Yes = 1, No = 0	0.471	0.491
	Undergraduate degree participant	Yes = 1, No = 0	0.118	0.425
Instrumental variable	Policy awareness	Awareness of the OVOUS program policy: Well-informed = 3, Somewhat informed = 2, Uninformed = 1	1.989	0.978

### Descriptive statistics

As shown in Table 1: First, among the respondents, 1502 respondents participated in the OVOUS project, accounting for approximately 58.9% of the total sample. Among them, 809 respondents participated in correspondence education, while 693 respondents participated in distance education. In addition, 1,050 respondents, or 41.1% of the sample, did not participate in the program. Second, the average proportion of agricultural income to total household income among respondents is 0.156, indicating that agricultural income from farming accounts for only a small portion of their total household income and that non-agricultural income is still one of the main sources of the respondents’ household economy. Non-agricultural income remains a major economic source for these farm households, reflecting the importance of the non-agricultural industries in diversifying rural household income. Third, the average age of the respondents is 46.971 years, with an average gender value is 0.757, indicating that the older males are the primary participants. Fourth, although the proportion of respondents participating in rural revitalization talent cultivation is relatively high, the mean policy awareness score is only 1.989, suggesting that the overall awareness of relevant policies remains limited across the sample. Fifth, the average education level of the respondents before participating in the OVOUS program is 3.51, indicating that most have an educational background at the junior high school or senior high school level.

Table 2 reports the differences in means for various variables between the treatment and control groups. Compared to the control group of respondents that did not participate in the OVOUS program, the treatment group exhibits significant difference across individual and household characteristics. At the individual level, participating farm households tend to have younger members, higher-level education, and higher proportion of village cadres. At the household level, participating farm households large cultivated land area and more family members engaged in migrant work. Additionally, farm households that participated in the OVOUS program show significantly higher agricultural income, with differences in means significant at the 1% statistical level. However, despite these significant differences, the data cannot definitively prove that these differences are entirely caused by the OVOUS program. To more rigorously examine the impact of the program on improving farm households’ agricultural income, further econometric analysis is required for validation.

**Table 2:** Between-group differences in variables.

Variable type	Variable name	Treatment group	Control group	Difference	t-value
Explained variable	Degree of part-time farming	0.196 (0.288)	0.099 (0.267)	0.099***	8.063
Individual characteristic	Age	39.363 (7.390)	57.871 (10.829)	–18.888***	–46.505
	Gender	0.725 (0.447)	0.804 (0.397)	–0.085***	–4.543
	Education level	4.539 (1.075)	2.035 (1.038)	2.649***	55.857
	Village cadres	0.598 (0.490)	0.217 (0.413)	0.389***	19.771
	Migrant worker experience	0.859 (0.348)	0.350 (0.477)	0.517***	28.099
Household characteristics	Family size	5.024 (1.623)	4.469 (1.986)	0.543***	6.913
	Largest surname in village	0.462 (0.498)	0.352 (0.478)	0.105***	4.975
	Number of laborers	3.054 (1.240)	2.345 (1.386)	0.704***	12.276
	Number of migrant workers	1.919 (1.373)	0.831 (1.113)	1.109***	20.592
	Cooperative membership	0.244 (0.429)	0.050 (0.217)	0.209***	13.645
	Online agricultural sales	0.135 (0.342)	0.014 (0.119)	0.124***	11.016
	Farmland transfer in	0.697 (0.459)	0.086 (0.280)	0.601***	36.378
	Cultivated land area	12.413 (62.754)	4.713 (14.450)	7.757***	3.740
Regional characteristics	Village terrain	1.965 (0.723)	2.589 (0.561)	–0.619***	–22.062
	Distance to county seat	25.102 (19.643)	29.974 (21.642)	–4.245***	–4.571
	Regional economic development level	9.586 (0.239)	9.576 (0.204)	0.010	1.093
Instrumental variable	Policy awareness	2.428 (0.891)	1.359 (0.717)	1.199***	58.869

Note: * , **, and *** indicate significance at the 10%, 5%, and 1% levels, respectively (applies to all tables below).

### Model construction

Due to the self-selection bias with farm households’ participation in rural revitalization talent cultivation, the analysis faces endogeneity issues. The decision to participate is endogenous and may involve selection bias. Specifically, farm households that choose to participate in the OVOUS program may already have higher agricultural income than average farm households. This self-selection implies that participating farm households may inherently possess advantages in attributes such as talent, education level, agricultural production techniques, and operational capacities. To address this issue, this study employs an Endogenous Switching Regression (ESR) model to overcome self-selection bias. Based on counterfactual analysis, the model explores the causal relationship between farm households’ participation in rural revitalization talent cultivation and their part-time farming, ensuring the reliability of the estimation results. The ESR model accounts for both observable and unobservable factors influencing individual decisions to participate and evaluates the average treatment effect under counterfactual scenarios, revealing the true impact of rural revitalization talent cultivation and farm households’ part-time farming.

The ESR model simultaneously estimates the following equations:


$$Y_{i}=\beta^{\prime }X_{i}+\delta^{\prime }T_{i}+\varepsilon_{i}$$ (1)

In Equation 1, $Y_{i}$ represents the degree of part-time farming among farm household; $X_{i}$ denotes individual, farm household, and location characteristics that influence part-time farming; $T_{i}$ is the decision variable for whether the farm household participates in rural revitalization talent cultivation ($T_{1}$ for participation, $T_{0}$ for non-participation); β' represents the coefficient to be estimated; and $\varepsilon_{i}$ represents the random error term.

The estimation of the ESR model involves two main stages: First, a behavioral equation is regressed to determine the likelihood of farm household participation in the rural revitalization talent cultivation; Second, an outcome equation is regressed to measure the effects of participation in the program and its various cultivation modes on part-time farming. Specifically, the ESR model estimates the following equations:

Stage 1: Behavioral equation (farm household participation in rural revitalization talent cultivation):


$$T_{i}=\gamma Z_{i}+\mu_{i}$$ (2)

Phase II

Outcome Equation 1 (treatment group, degree of part-time farming for non-participating farm households):


$$Y_{1}=\eta_{1}X_{i}+\nu_{1}{}_{i}$$ (3)

Outcome Equation 2 (control group, degree of part-time farming for non- participating farm households):


$$Y_{2}=\eta_{2}X_{i}+\nu_{2i}$$ (4)

In Equation 2, $T_{i}$ represents whether a farm household participates in rural revitalization talent cultivation, defined as a binary variable; $Z_{i}$ is a set of factors influencing the farm household’s decision to participate in the program; and $\mu_{i}$ is the error term, capturing unobservable factors.

In Equations 3 and 4, $Y_{1}$ and $Y_{2}$ denote the degree of part-time farming for the treatment and control groups, respectively; $\eta_{1}$ and $\eta_{2}$ are parameters to be estimated; $X_{i}$ is a set of factors influencing the degree of part-time farming; and $\nu_{1i}$ and $\nu_{2i}$ are the error terms for the outcome equations.

The ESR model provides of the differentiated effects of various factors on the degree of part-time farming for the treatment and control groups. To assess the overall impact of the program on the agricultural income for participating farm households, the average treatment effect is calculated using the ESR model coefficients:

Average treatment effect on treated (ATT):


$$ATT=E\left(Y_{i}{}_{1}\text{|}T_{i}=1\right)-E(Y_{i}{}_{0}|T_{i}=1)$$ (5)

Average treatment effect on the untreated (ATU):


$$ATU=E\left(Y_{i}{}_{1}\text{|}T_{i}=0\right)-E(Y_{i}{}_{0}|T_{i}=0)$$ (6)

In summary, the average of ATT and ATU is used in this study to evaluate the average treatment effect of rural revitalization talent cultivation on the degree of part-time farming among farm households.

## Results analysis and discussion

### ESR model estimation results and analysis

Table 3 presents the ESR model estimation results regarding the impact of rural revitalization talent cultivation on the degree of part-time farming among farm households. From the regression results of the selection equation, several factors significantly influence farmers’ participation in rural revitalization talent cultivation. First, the age of farmers negatively affects their likelihood of participation, consistent with previous research [30]. As farmers age, they face greater challenges in adapting to new technologies, concepts and opportunities brought about by social challenges. Moreover, older farmers are more inclined to maintain the status quo, lacking the motivation to adopt new practices. Second, gender significantly influences participation decisions, with female farmers being more likely to participate in rural revitalization talent cultivation. This may be attributed to the traditional division of labor in rural households, where women often bear greater responsibilities in agricultural production and household chores. As a result, women are more motivated to enhance their agricultural skills through training to support household production. Furthermore, education level has a significant positive effect on farmers’ participation, indicating that higher education levels make it easier for farmers to adapt to and engage in rural revitalization talent cultivation. Similarly, migrant work experience significantly increases participation willingness. This is likely because such experience enhances famers’ human, social and financial capital, making them more aware of the potential benefits of agricultural modernization. Lastly, the number of migrant workers and cultivated land area significant affect participation. Households with more migrant workers tend to have lower demand for agricultural skills, as more labor is devoted to non-agricultural sectors. In contrast, households with larger cultivated land areas have a higher demand for agricultural skills and are more inclined to participate in training to reduce production risks and stabilize agricultural income. Thus, household with fewer migrant workers and larger cultivated land areas are more likely to participate in rural revitalization talent cultivation.

**Table 3:** Benchmark regression results.

Variable	Selection equation		Outcome equation			
Treatment group		Control group	
Coefficient	Std. Error	Coefficient	Std. Error	Coefficient	Std. Error
Age	−0.102^***^	0.009	0.002^**^	0.001	−0.003^***^	0.001
Gender	−0.337 *	0.189	0.055^***^	0.015	0.087^***^	0.021
Educational level	0.691^***^	0.073	0.018^***^	0.006	−0.004	0.008
Village cadres	0.516^***^	0.186	0.008	0.017	−0.004	0.023
Migrant worker experience	0.494^**^	0.204	−0.238^***^	0.019	−0.106^***^	0.019
Family size	0.112^**^	0.056	−0.007	0.005	−0.023^***^	0.005
Largest surname in village	0.181	0.147	−0.019	0.013	−0.002	0.017
Number of laborers	−0.264^***^	0.092	0.042^***^	0.008	−0.009	0.009
Number of migrant workers	0.517^***^	0.081	−0.043^***^	0.007	−0.003	0.009
Cooperatives membership	0.746^***^	0.241	0.065^***^	0.016	0.048	0.037
Online agricultural sales	0.741^***^	0.347	0.120^***^	0.019	−0.021	0.067
Actual cropland area	−0.001	0.001	0.001^***^	0.000	−0.001	0.001
Village terrain	−0.700^***^	0.112	−0.016 *	0.009	−0.009	0.014
Distance to county seat	−0.003	0.003	0.001	0.001	0.001 *	0.000
Regional economic development level	0.759^***^	0.315	0.004	0.027	−0.039	0.039
Policy awareness	0.410^***^	0.082				
rho_1			0.558^***^	0.142		
lnσ1			−1.415^***^	0.019		
rho_0					0.163	0.121
lnσ0					−1.374^***^	0.022
LR	9.780^***^					
Log likelihood	−210.902					
N	2552		1502		1050	

From the regression results of the outcome equation, the mechanisms influencing the degree of part-time farming differ between the treatment group (participants) and the control groups (non-participants). For non-participants in the control group, male farmers have a more pronounced inhibitory effect on their part-time farming compared to female farmers, indicating that male farmers tend to focus more on agricultural production. Additionally, the distance from the village to the county seat negatively affects part-time farming, with farm households closer to the county benefiting more from the radiating effects of agricultural modernization. In the treatment group, several variables significantly influence the degree of part-time farming. Higher education levels enhance farmers’ ability to access diverse market information and apply advanced techniques, giving them an advantage in agricultural production and enabling them to increase agricultural income, thereby reducing part-time farming. Furthermore, cultivated land area is negatively correlated with part-time farming, indicating that farm households participating in rural revitalization talent cultivation reduce part-time farming by expanding their production scale. Meanwhile, for non-participants group, the degree of part-time farming shows a positive correlation with age, suggesting that traditional farming methods may fail to meet the needs of ageing farmers. In contrast, for participants in the treatment group, age has a negative correlation with part-time farming, indication that rural revitalization talent cultivation helps farmers overcome age-related constrains on agricultural production. This finding is particularly significant for addressing the challenges posed by an aging rural population. In addition, topography also affects the degree of part-time farming. Mountainous regions exhibit a positive relationship with part-time farming, indicating that natural conditions in these areas drive farmers to rely more heavily on non-agricultural income. Finally, online agricultural sales have a significant negative effect on part-time farming in the treatment group, highlighting the potential of rural revitalization talent cultivation to enhance farmers’ e-commerce capabilities and storage and preservation techniques. This enables farmers to expand their agricultural product sales channels, thereby reducing their reliance on non-agricultural income. This finding further underscores the importance of digital technology in promoting rural economic development.

### Analysis of treatment effects

The study shows that rural revitalization talent cultivation has a significant negative impact on the degree of part-time farming among farm household. Specifically, farm households participating in rural revitalization talent cultivation have a significantly lower degree of part-time farming compared to those that do not participate. To more accurately evaluate this impact, this study employs a fact-based and computational analysis to control for sample selection bias, quantifying the differences in the degree of part-time farming between the treatment and control groups. The detailed results are presented in Table 4. First, based on the counterfactual degree of part-time farming for the treatment group in the absence of participation in rural revitalization talent cultivation, ATT is calculated as 0.056 using Equation 5. This indicated that if farm households participating in rural revitalization talent cultivation were in a counterfactual situation where they did not participate, their degree of rural revitalization talent cultivation in reducing the degree of part-time farming among farm households. Second, for the counterfactual degree of part-time farming for the control group, assuming they had participated in rural revitalization talent cultivation, ATU is calculated as 0.125 using Equation 6. This suggests that if non-participating households had participated, their degree of part-time farming would have decreased by 0.125. Finally, by comparing the overall degree of part-time farming between the treatment and control groups, ATE is calculated as 0.084. This means that farm households participating in rural revitalization talent cultivation have a degree of part-time farming that is 0.084 lower than those that do not participate. These results confirm that rural revitalization talent cultivation significantly reduces the degree of part-time farming among farm households, thereby validating Hypothesis 1. Therefore, rural revitalization talent cultivation plays an important role in optimizing rural labor allocation and enhancing the stability of agricultural income.

**Table 4.** Average treatment effects of rural revitalization talent cultivation on part-time farming.

	Treatment group	Control group	ATT	ATU	ATE
Treatment group	0.196 (0.158)	0.142 (0.096)	0.056*** (0.005)		
Control group	0.224 (0.148)	0.099 (0.086)		0.125*** (0.005)	
All farm households	0.208 (0.151)	0.124 (0.094)			0.084*** (0.003)

### Heterogeneity analysis

#### Educational level.

For farm households, the treatment effect of rural revitalization talent cultivation on their degree of part-time farming exhibits significant heterogeneity based on education level, as shown in Table 5. According to the treatment effect results, talent cultivation at both college’s and undergraduate’s education levels significantly reduce the degree of part-time farming among farm households, but the effect of undergraduate’s education is more pronounced. Specifically, the ATT value for farm households participating in college’s education is 0.056, while for those participating in undergraduate’s education, the ATT value is 0.075. This is indicates that farm households receive undergraduate’s education experience a greater reduction in part-time farming compared to those with college’s education. The notable advantage of bachelor’s education in reducing part-time farming may be attributed to the depth of learning and the enhancement of comprehensive skills it provides. Higher-level education fosters stronger innovation capabilities and problem-solving skills [31], enabling farm households to better address risks and uncertainties in agricultural production. These findings validate Hypothesis 2, namely that higher-level of education in rural revitalization talent cultivation have a more significant effect on reducing the degree of part-time farming.

**Table 5:** Group treatment effects of rural revitalization talent cultivation on part-time farming.

			Treatment group	Control group	ATT	ATU	ATE
Educational level	College education	Process group	0.199	0.143	0.056^***^		0.087^***^
		Control subjects	0.223	0.099		0.123^***^	
	Undergraduate education	Process group	0.193	0.118	0.075^***^		0.202^***^
		Control subjects	0.337	0.099		0.238^***^	
Cultivation method	Correspondence education	Process group	0.206	0.127	0.079^***^		0.187^***^
		Control subjects	0.369	0.099		0.269^***^	
	Distance education	Process group	0.188	0.142	0.046^***^		0.102^***^
		Control subjects	0.239	0.099		0.139^***^	

#### Cultivation mode.

The impact of rural revitalization talent cultivation on the degree of part-time farming among farm households also varied significantly with different cultivation modes, as shown in Table 5. The treatment effect results indicate that both correspondence education and distance education significantly reduce the degree of part-time farming, but correspondence education is more effective. Specifically, the ATT value for households participating in correspondence education is 0.079, while for those participating in distance education, the ATT value is 0.046. This demonstrates that correspondence education is more effective than distance education in helping farm households reduce their degree of part-time farming. The significant advantage of correspondence education may stem from its systematic course design and the integration of practical components. Compared to distance education, which relies on self-directed learning and digital resources [32], correspondence education typically provides more face-to-face teaching and regular guidance, allowing farm households to gain a deeper understanding of complex agricultural knowledge. Furthermore, correspondence education often includes practical courses, such as field experiments and on-site guidance, which enable farm households to directly apply the skills they have learned to agricultural production. In contrast, the lack of structured practical support in distance education may limit its effectiveness in reducing the degree of part-time farming. These findings validate Hypothesis 3, namely that correspondence education is more effective than distance education in reducing the degree of part-time farming among farm households.

### Robustness tests

#### Propensity matching score method (PSM).

To verify the robustness of the results, this section employs the Propensity Score Matching (PSM) method to re-estimate the suppressing effect of rural revitalization talent cultivation on farm household part-time farming. Specifically, various matching methods, including nearest-neighbor matching, radius matching, kernel matching, and stratified matching, were used to obtain the estimated results, as shown in Table 6. The ATT value obtained through nearest-neighbor matching is 0.115 and is significant at the 1% statistical level. The indicates that the probability of reduced part-time farming for farm households participating in rural revitalization talent cultivation is 11.5% higher than the counterfactual scenario where they did not participate. Similar significant results were found using other matching methods, such as kernel matching and stratified matching, confirming that rural revitalization talent cultivation has a robust effect on reducing part-time farming among farm households.

**Table 6:** ATT results from different propensity score matches.

Matching method	Treatment group	Control group	ATT	Standard error	t-value
Nearest-neighbor matching	1502	50	0.115^**^	0.048	2.415
Radius matching	1502	207	0.130^**^	0.108	1.198
Kernel matching	1502	207	0.125^**^	0.049	2.563
Stratified matching	1502	211	0.127^**^	0.059	2.166

Note: The radius for radius matching is set at 0.005.

#### IV-Tobit model.

The IV-Tobit model was further employed to verify the robustness of the baseline regression results, and the estimation results are presented in Table 7. Column (1) of Table 7 reports the Tobit model results, provided for reference purposes, while Column (2) focuses on the IV-Tobit regression results. First, the Wald test indicates that the null hypothesis of “α=0” for exogeneity can be rejected, confirming the presence of endogenous variables in the model. Specifically, the first-stage regression results show that the level of policy awareness significantly increases the likelihood of farm household participation in rural revitalization talent cultivation. The second-stage regression results indicate that rural revitalization talent cultivation has a significant positive effect on reducing part-time farming, with a coefficient of 0.512 (significant at the 5% level). These findings further support the results of the baseline regression model, demonstrating that rural revitalization talent cultivation has a significant effect in reducing the degree of part-time farming among farm households and confirming the validity of policy awareness as an instrumental variable.

**Table 7:** Results of IV-Tobit model estimation.

Variable	Tobit	IV-Tobit	
First stage	Second stage
Rural revitalization talent cultivation	0.144^***^		0.512^**^
Policy awareness		0.056^***^	
Control variables	Control	Control	Control
N	2552	2552	2552
R^2^		0.77	
F-value		501.15	
Wald chi^2^		513.09	

## Conclusions and policy recommendations

The realization of rural revitalization is inseparable from the development of a strong agricultural talent pool, especially as agricultural modernization continues to progress. Using Jiangxi Province’s OVOUS program as an example, this study empirically analyzed the impact of rural revitalization talent cultivation on farm household part-time farming and reached the following main conclusions:

1. Rural revitalization talent cultivation significantly reduces the degree of part-time farming among farm households. Robustness and endogeneity tests yielded consistent results. Farm households participating in talent cultivation programs exhibit significantly lower levels of part-time farming compared to non-participating households, indicating that talent cultivation helps farmers focus more on agricultural activities, effectively suppresses part-time farming.
2. Higher-level education have a more pronounced effect on reducing the degree of part-time farming. Farm households receiving undergraduate’s education demonstrate substantial improvements in agricultural skills, innovation capacity, and market adaptability, leading to greater reduction in part-time farming.
3. Correspondence education is more effective in reducing part-time farming. Compared to distance education, correspondence education leverages systematic teaching materials and regular in-person guidance to deepen farmers’ agricultural knowledge and practical skill. This enables farmers to better apply what they have learned, thereby improving agricultural productivity and reducing part-time farming.

Based on the above conclusions, this paper proposes the following policy recommendations.

1. Strengthen rural revitalization talent cultivation efforts. Further promote and deepen the implementation of rural revitalization talent programs, with particular emphasis on policy support for underdeveloped and impoverished rural areas. Ensure more farm households have access to education and training opportunities, thereby improving their overall knowledge and skill levels. Additionally, tailor training programs to regional characteristics and industrial needs to enhance the specificity and effectiveness of talent cultivation.
2. Enhance education level and content. Encourage farm households to pursue education at the undergraduate’s level or higher. Simultaneously, optimize curriculum design by incorporating practical courses on agricultural technology, market operations, and management. This will equip farmers with the skills needed to address market fluctuations and environmental challenges, thereby enhancing agricultural productivity and income stability.
3. Optimize education and training methods. While maintaining the flexibility of distance education, increase the proportion of correspondence education. Combine in-person instruction with practical courses to enhance the effectiveness of education and training programs. Additionally, strengthen cooperation with agricultural extension agencies and farmers’ cooperatives to provide more on-site practices and technical guidance, enabling farmers to more efficiently transform theoretical knowledge into productive capabilities.
4. Enhance policy awareness and support measures. Improve farmers’ awareness of rural revitalization talent cultivation policies by promoting these programs through various channels, such as village bulletin boards, television broadcasts, and social media. Simplify participation processes and provide supporting policies, including tuition subsidies, low-interest loans, and technical assistance, to reduce the financial burden of education and training for farm households. These measures will further encourage farmers to actively participate in such programs.

By refining and implementing these policy recommendations, farmers can be better guided to participate in rural revitalization talent cultivation, thereby enhancing agricultural productivity, optimizing rural labor allocation, and advancing comprehensive rural revitalization.
